# Interest in the Use of Herbal Supplements to Close the Treatment Gap for Hazardous Alcohol Use Among Men Who Have Sex With Men: Secondary Analysis of a Cross-Sectional Study

**DOI:** 10.2196/60370

**Published:** 2024-10-29

**Authors:** Christopher Hernandez, Christopher Rowe, Janet Ikeda, Justine Arenander, Glenn-Milo Santos

**Affiliations:** 1 David Geffen School of Medicine University of California Los Angeles Los Angeles, CA United States; 2 Center on Substance Use and Health San Francisco Department of Public Health San Francisco, CA United States; 3 Department of Community Health Systems University of California San Francisco San Francisco, CA United States

**Keywords:** alcohol use disorder, herbal supplements, HIV, herbal, supplement, alcohol, alcoholic, alcohol use, alcohol consumption, cross-sectional study, California, USA: binge drinking, alcohol dependence, men, social, clinical, logistic regression, drinking

## Abstract

**Background:**

Hazardous alcohol consumption is highly prevalent for men who have sex with men (MSM). The 4 treatments currently approved by the Food and Drug Administration for alcohol use are reaching an alarmingly low percentage of people who would benefit from a reduction in their alcohol use. There is increasing interest in alternative methods of treatment, such as herbal supplements, to address hazardous drinking. However, research on the acceptability of alternative pharmacotherapies among MSM remains limited.

**Objective:**

We examined the prevalence and correlates of expressing interest in using herbal supplements for alcohol treatment among MSM with hazardous alcohol consumption.

**Methods:**

We conducted a secondary data analysis from a cross-sectional study of MSM who use alcohol, conducted from March 2015 to July 2017 in San Francisco, California, to assess the overall prevalence of interest in using herbal supplements to help reduce alcohol consumption. Associations between expressing interest in herbal supplements and demographic, social, and clinical characteristics were examined using bivariate and multivariable logistic regression models.

**Results:**

One-third (66/200, 33%) of the participants expressed interest in an herbal supplement for reducing alcohol consumption. In the multivariable analyses, weekly binge drinking (adjusted odds ratio [aOR] 2.85, 95% CI 1.17-6.93), interest in abstaining from alcohol use (aOR 5.04, 95% CI 1.46-17.40), higher severity of alcohol dependence score (aOR 1.22, 95% CI 1.04-1.41), and interest in naltrexone (aOR 3.22, 95% CI 2.12-4.91) were independently associated with higher odds of being interested in using an herbal supplement to reduce alcohol consumption, adjusting for age, race or ethnicity, and education.

**Conclusions:**

We found that MSM who have hazardous drinking habits, more severe alcohol dependence, and interest in pharmacotherapy were more likely to express interest in using an herbal supplement for reducing alcohol consumption. To our knowledge, this is the first study that has evaluated correlates of interest in herbal supplements for alcohol use among MSM. As researchers implement novel alcohol treatment studies, they should focus on recruitment efforts among MSM with a motivation to reduce their alcohol use patterns.

## Introduction

The prevalence of hazardous alcohol consumption, which includes binge drinking (5 or more standard drinks for men in 1 occasion), is high among men who have sex with men (MSM) in the United States. Binge drinking approached 51% among MSM in the 2015 National Survey on Drug Use and Health [1]. This pattern of alcohol use poses significant public health and economic burdens, with associated costs of more than US $250 billion in 2010 alone [2]. Alcohol use is a significant risk factor for many poor health outcomes, including physical and mental ramifications that range from end organ damage to various cancers and exacerbation of depression and anxiety. Concerningly, problematic event-level alcohol use among at-risk populations is strongly associated with HIV infection [3]. Binge drinking, in particular, has been found to be associated with condomless anal intercourse among MSM, likely by impairing safety risk assessment [4,5]. Effective tackling of the high prevalence of binge drinking among MSM would have far-reaching positive effects on both individual and community-wide health outcomes.

Behavioral and pharmacological interventions for alcohol use are not effectively reaching individuals with hazardous alcohol consumption. For example, it is estimated that less than 10% of patients with alcohol use disorders have ever received a prescription for a medication for alcohol treatment in outpatient settings [6], with less data available in other samples. This utilization of current alcohol treatment strategies also remains low among convenience samples of MSM groups that have alcohol-mediated HIV risk [7]. For example, the SEEDS study, a cross-sectional study among MSM who use alcohol in San Francisco, California, found that only 6.0% of MSM who use alcohol have ever used medication for alcohol treatment [8]. Often cited as self-reported reasons for not using medications for alcohol treatment include concerns regarding drug side effects and stigma associated with taking a pharmacologic agent to treat alcohol use [9,10]. These findings suggest a need for alternative treatments that are significantly more acceptable among MSM who require intervention for their alcohol use.

Herbal and natural supplements may represent a promising alternative approach to treatment modalities. Herbal remedies for alcohol abuse have been used for medicinal purposes in East Asia for centuries [11]. In recent years, there has been increasing interest in the use of herbal supplements as alcohol treatment among researchers. For example, there is an ongoing placebo-controlled study of the supplement N-acetylcysteine as a treatment for alcohol use disorder among veterans with traumatic brain injury [12]. An ongoing intervention study is being conducted to determine the efficacy of the herbal supplement kudzu in reducing alcohol consumption [13]. Kudzu has shown promise in being an efficacious treatment for alcohol consumption in several studies [14,15]. Not much is known about the acceptability of herbal supplements to treat alcohol use among MSM. This study aimed to examine the prevalence and correlates of interest in using herbal supplements for alcohol treatment among MSM with hazardous alcohol consumption.

## Methods

### Study Design

This secondary analysis used data from the SEEDS study conducted from March 2015 to July 2017 in San Francisco, California. SEEDS was a cross-sectional study that used respondent-driven sampling (RDS) to assess alcohol use patterns and correlates of hazardous use in a diverse sample of MSM who use alcohol, and the methods for the main study are described elsewhere [8]. The study recruited 252 participants meeting the following inclusion criteria: (1) used alcohol at least once in the past year, (2) had sex with men, (3) were at least 18 years of age, (4) resided in the San Francisco Bay Area, and (5) had been assigned male sex at birth and identified as male.

### Measures

The SEEDS study assessed alcohol use and dependence among participants with the Alcohol Use Disorders Identification Test (AUDIT) and Severity of Dependence Scale [16,17]. Participants were also asked about their demographic, clinical, and behavioral history. Questions assessing interest in reducing alcohol consumption, interest in pharmacotherapy, and interest in herbal supplements had 4 response options: not interested, somewhat interested, moderately interested, and extremely interested. For this analysis, response options were dichotomized, such that those reporting moderate to extreme interest in using an herbal supplement were compared with those not interested or somewhat interested. This analysis was restricted to participants with hazardous alcohol use, as determined by an AUDIT score of 8 and above. This restriction limits the study sample to individuals with alcohol use that is considered high risk for harmful outcomes and for whom the recommended screening for alcohol use interventions [18].

### Analysis

The restriction on the subset of participants with hazardous alcohol consumption in this secondary data analysis precludes our ability to conduct weighted analyses derived from recruitment patterns in the entire sample from the parent study [19]. Therefore, we analyzed the data without RDS sampling weights, as appropriate for subgroup analyses. We examined the associations between the outcome interest in herbal supplements and potential demographic, social, and clinical correlates with bivariate logistic regression models. We conducted a multivariable model to test the associations between interest in herbal supplements and correlates that were significant (*P*≤.25) in the bivariate analysis using a backward stepwise model building [20,21].

### Ethical Considerations

The study procedures for this paper were reviewed and approved by the University of California San Francisco institutional review board (approval 14-14481). The authors have permission to use the data used in this analysis.

## Results

### Demographics and Alcohol Use Prevalence

The original dataset contained 252 MSM who use alcohol. After restricting the sample to those with hazardous alcohol use, 200 participants remained. The restricted sample reflected a diverse group of MSM, where 31.5% (63/200) were White, 32% (34/200) were African American/Black, 12.5% (25/200) were Asian or Pacific Islander, 16.5% (33/200) were Hispanic/Latino, and 7.5% (15/200) identified as mixed race. Moreover, 55.5% (111/200) of the participants scored 3 or more on the Severity of Dependence Scale. Furthermore, 56.5% (113/200) of the participants self-reported weekly binge drinking.

### Alcohol Reduction Goals

Goals related to reducing alcohol use varied among participants, with 27.5% (55/200) reporting no specified target, 48% (96/200) reporting interest in reducing their use, and 21% (42/200) expressing the desire to abstain from alcohol consumption altogether. Moreover, 57.5% (115/200) of the participants expressed interest in reducing alcohol consumption. In total, 33% (66/200) of the participants expressed interest in an herbal supplement to reduce their alcohol use.

### Bivariate Analysis

As shown in the 2 rightmost columns of Table 1, interest in herbal supplements was significantly correlated with AUDIT score, Severity of Dependence Scale score, specified goal, interest in reducing alcohol, interest in pharmacotherapy, interest in naltrexone, previous attempts to stop alcohol use, participation in alcohol intervention, alcohol use during sex, substance use in the last 6 months, and participation in a drug treatment program (all *P*<.05).

**Table 1:** Bivariate logistic regression for interest in herbal supplements for alcohol treatment and correlates among San Francisco men who have sex with men from a cross-sectional study.

Characteristic			No interest (n=134)		Interest (n=66)		Total (n=200)		Odds ratio (95% CI)		*P* value	
Age (years), mean (SD)			41 (12.62)		41 (11.71)		41 (12.3)		1.0 (0.97-1.02)		.97	
**Race and ethnicity, n (%)**												
	White	41 (30.60)		22 (33.33)		63 (31.5)		Reference^a^				
	Black/African American	48 (35.82)		16 (24.24)		64 (32)		0.62 (0.28-1.34)		.22		
	Asian/Pacific Islander	18 (13.43)		7 (10.61)		25 (12.5)		0.72 (0.26-2.00)		.53		
	Hispanic/Latino	20 (14.93)		13 (19.70)		33 (16.5)		1.21 (0.51-2.89)		.67		
	Mixed race	7 (5.22)		8 (12.12)		15 (7.5)		2.13 (0.68-6.65)		.19		
**School/education (no interest: n=133; interest: n=66; total: n=199), n (%)**												
	High school or less	42 (31.58)		21 (31.82)		63 (31.66)		Reference				
	Some college	44 (33.08)		24 (36.36)		68 (34.17)		1.09 (0.52-2.24)		.81		
	Bachelor’s or postgraduate studies	47 (35.34)		21 (31.82)		68 (34.17)		0.89 (0.43-1.86)		.76		
**Income (US $; no interest: n=128; interest: n=66; total: n=194), n (%)**												
	0-9999	45 (35.16)		27 (40.91)		72 (37.11)		Reference				
	10,000-19,999	24 (18.75)		9 (13.64)		33 (17.01)		0.63 (0.25-1.54)		.31		
	20,000-39,000	19 (14.84)		15 (22.73)		34 (17.53)		1.32 (0.57-3.01)		.52		
	40,000-74,999	14 (10.94)		5 (7.58)		19 (9.79)		0.60 (0.19-1.84)		.37		
	75,000+	26 (20.31)		10 (15.15)		36 (18.56)		0.64 (0.27-1.53)		.32		
**AUDIT^b^ zone, n (%)**												
	Zone 2	77 (57.46)		19 (28.79)		96 (48)		Reference				
	Zone 3	25 (18.66)		11 (16.67)		36 (18)		1.78 (0.75-4.25)		.19		
	Zone 4	32 (23.88)		36 (54.55)		68 (34)		*4.55 (2.28-9.11)* ^c^		*<.001*		
**Severity of Dependence Scale score of >3, n (%)**												
	Score above 3	62 (46.27)		49 (74.24)		111 (55.5)		*1.24 (1.24-1.37)*		*<.001*		
	Reports any binge drinking	130 (97.01)		64 (96.97)		194 (97)		0.98 (0.18-5.52)		.99		
	Reports weekly binge	65 (48.51)		48 (72.73)		113 (56.5)		*2.83 (1.49-5.36)*		*.001*		
	Reports daily binge	22 (16.42)		17 (25.76)		39 (19.5)		1.76 (0.86-3.62)		.12		
**Goal** **(no interest: n=129; interest: n=64; total: n=193), n (%)**												
	No goal specified	44 (34.11)		11 (17.19)		55 (28.5)		Reference				
	Reduction	68 (52.71)		28 (43.75)		96 (49.74)		1.65 (0.74-3.64)		.22		
	Abstinence	17 (13.18)		25 (39.06)		42 (21.76)		*5.88 (2.38-14.52)*		*<.001*		
**Interest in reducing alcohol, n (%)**												
	Not interested	38 (28.36)		1 (1.52)		39 (19.5)		Reference				
	Somewhat	66 (49.25)		14 (21.21)		80 (40)		*8.06 (1.02-63.73)*		*.048*		
	Moderately	24 (17.91)		17 (25.76)		41 (20.5)		*26.92 (3.36-215.57)*		*.002*		
	Extremely	6 (4.48)		34 (51.52)		40 (20)		*215.33 (24.67-1880.25)*		*<.001*		
**Interest in pharmacotherapy, n (%)**												
	Not interested	80 (59.7)		5 (7.58)		85 (42.5)		Reference				
	Somewhat	47 (35.07)		15 (22.73)		62 (31)		*5.11 (1.74-14.95)*		*.003*		
	Moderately	4 (2.99)		20 (30.3)		24 (12)		*80 (19.67-325.43)*		*<.001*		
	Extremely	3 (2.24)		26 (39.39)		29 (14.5)		*138.67 (31.00-620.33)*		*<.001*		
**Interest in naltrexone, n (%)**												
	Not interested	53 (39.55)		6 (9.09)		59 (29.5)		Reference				
	Somewhat	51 (38.06)		14 (21.21)		65 (32.5)		2.42 (0 .87-6.79)		.09		
	Moderately	21 (15.67)		19 (28.79)		40 (20)		*7.99 (2.80-22.79)*		*<.001*		
	Extremely	9 (6.72)		27 (40.91)		36 (18)		*26.5 (8.54-82.21)*		*<.001*		
Tried to stop drinking? n (%)			95 (70.9)		57 (86.36)		152 (76)		*2.6 (1.17-5.76)*		*.02*	
Previous alcohol intervention? n (%)			40 (29.85)		34 (51.52)		74 (37)		*2.49 (1.36-4.59)*		*.003*	
**Type of alcohol Intervention, n (%)**												
	Behavioral (CBT^d^ or other counseling)	19 (14.18)		13 (19.7)		32 (16)		1.48 (0.68-3.23)		.32		
	Medication	4 (2.99)		9 (13.64)		13 (6.5)		*5.13 (1.52-17.35)*		*.009*		
	Mutual support	28 (20.9)		25 (37.88)		53 (26.5)		*2.31 (1.21-4.42)*		*.01*		
	Something else	8 (5.97)		5 (7.58)		13 (6.5)		1.29 (0.41-4.11)		.67		

^a^Reference group.

^b^AUDIT: Alcohol Use Disorders Identification Test.

^c^Odds ratios in italics indicate *P*<.05.

^d^CBT: cognitive behavioral therapy.

### Multivariate Analysis

**Figure 1:**
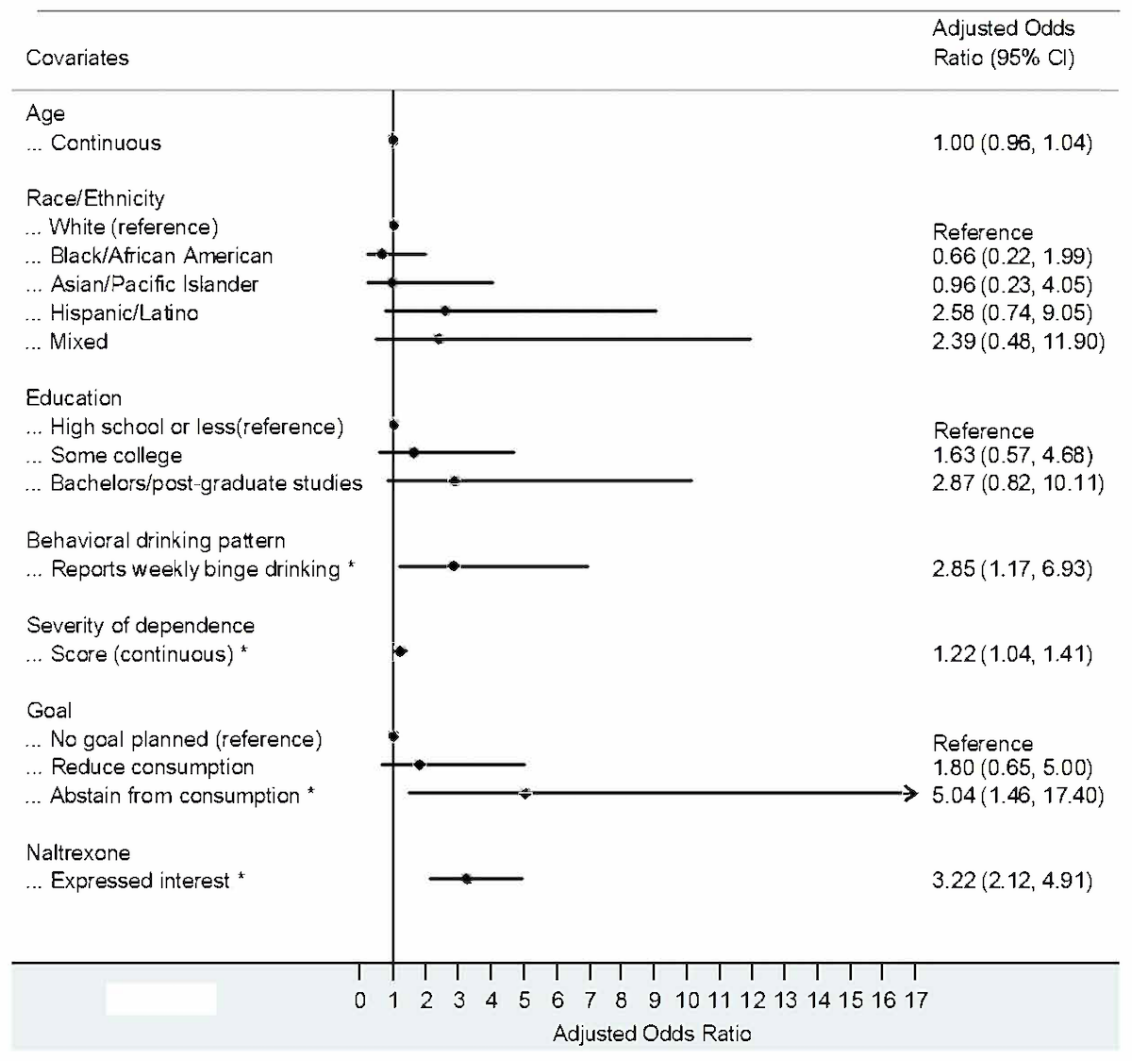
Multivariable logistic regression model results: interest in an herbal supplement among San Francisco men who have sex with men with hazardous alcohol use from a cross-sectional study. *P<.05.

Weekly binge drinking (adjusted odds ratio [aOR] 2.85, 95% CI 1.17-6.93), interest in abstaining from alcohol use (aOR 5.04, 95% CI 1.46-17.40), interest in naltrexone (aOR 3.22, 95% CI 2.12-4.91), and higher severity of alcohol dependence scores (aOR 1.22, 95% CI 1.04-1.41) were independently associated with higher odds of being interested in an herbal supplement to reduce alcohol consumption, after adjusting for age, race or ethnicity, and education (Figure 1).

## Discussion

### Principal Findings

In this sample of MSM who use alcohol with hazardous drinking patterns, we found that interest in the use of herbal supplements is high (66/200, 33%) among hazardous drinkers and associated with specific clinical and behavioral characteristics. Specifically, we observed that interest in an herbal supplement was significantly correlated with the goal of abstinence, interest in naltrexone, weekly binge drinking, and higher Severity of Dependence Scale score. These findings suggest that a population with high rates of hazardous drinking may find herbal supplements as an option to reduce their alcohol use.

We found that interest in using an herbal supplement to reduce alcohol use was associated with having more severe alcohol dependence and weekly binge drinking. Studies have shown a positive correlation between increasing alcohol dependence and aggregate alcohol-related problems [22,23]. We speculate that the negative consequences of alcohol use are a major motivating factor for interest in a broader range of approaches for alcohol use reduction, including herbal supplements. Research suggests that as negative consequences of alcohol use become more frequent and severe, individuals may be more willing to seek treatment and request help [24]. It is conceivable that a significant portion of participants reporting greater severity of dependence and frequency of binge drinking have experienced more persistent alcohol-related adverse effects, which may enhance interest and openness to engaging in a broader range of interventions, including alternative treatment modalities such as herbal supplements. In addition, we observed a significant association between interest in naltrexone for alcohol treatment and interest in an herbal supplement in this study. It is plausible that those familiar with existing pharmacotherapies such as naltrexone are actively exploring existing treatments for alcohol use and, therefore, may be more open to alternative treatment modalities as well.

Compared with those who did not have a specified goal, those who wanted to abstain from alcohol were more likely to express interest in an herbal supplement in the multivariable model. This finding is broadly consistent with the transtheoretical model that describes the stages of change, which posits that those who wish to abstain are more likely to be beyond the precontemplation stage of alcohol use reduction than those without a clear goal [25]. The action stage is hypothesized to be the point where patients are thought to be most receptive to treatment options, a period when physicians and interventionists are encouraged to provide options available to the patient [26]. This may also explain why participants with a high interest in naltrexone were also interested in herbal supplements. Individuals with the goal of abstinence goal in the action stage may have also been directed to information regarding treatment options either by a provider or through their own interest. Should ongoing herbal studies be shown efficacious [12,13], MSM who express a desire for abstinence should be informed and counseled on the availability of herbal supplements.

### Limitations

A limitation of our study is that all data are self-reported, which are subject to social desirability and recall biases. Social desirability bias, especially concerning substance use, could lead to underreporting. To minimize these concerns, we used self-administered computer assessments, which have been shown to reduce underreporting [27]. In addition, recall bias could limit the accuracy of answers to questions that asked about behaviors in the past. To enhance statistical power, our study dichotomized our outcome for interest in herbal supplements, which might lead to an oversimplification of the nuanced nature of participants’ attitudes and precludes use from distinguishing between very interested individuals and those with less considerable interest. In addition, our study is cross-sectional in nature, which precludes our ability to make any causal inferences. Moreover, all participants were restricted to a specific population, MSM living in San Francisco who had hazardous alcohol use, which could limit the generalizability of our findings. In addition, as noted in the *Methods* section, because we restricted our sample to those reporting hazardous alcohol use, we were unable to adjust for RDS sampling weights. Hence, our findings should be interpreted with caution, and we are not able to make any assumptions regarding the representativeness of our sample. In addition, while we did not observe differences in acceptability between racial and ethnic minority groups, the smaller samples across these groups may have precluded our ability to detect significant associations, as well as explore interactions between race or ethnicity and correlates of interest for herbal supplements. Furthermore, we recognize the wide confidence intervals in some of our point estimates (eg, goals of abstaining alcohol consumption), which reflect high imprecision and should therefore be interpretated with caution. Future studies among larger and more diverse samples of alcohol users are needed to confirm how our findings translate to other populations. However, despite these limitations, this study is valuable in that it encompasses a diverse group of MSM with hazardous alcohol use who may be at heightened risk for HIV due to their alcohol use. MSM who use alcohol is an important group to reach out to for novel or alternative interventions actively.

### Conclusions

This study highlights the acceptability of an alternative treatment for alcohol use and helps elucidate the characteristics of MSM motivated to address their hazardous drinking with an herbal supplement. Given the acceptability for herbal supplements, future research should continue to explore the efficacy and safety of herbal supplements as a treatment for alcohol use, particularly in diverse subpopulations beyond MSM. Furthermore, if studies determine their efficacy, efforts to integrate treatments such as herbal supplements into existing treatment frameworks, especially for patients who are either unwilling or unable to use conventional pharmacotherapy, might be worthwhile. With the low uptake of existing pharmacotherapy for alcohol use, alternative treatments may present an important and acceptable strategy in the armamentarium of treatment approaches for consumption. Ultimately, our findings support the potential of alternative therapies for alcohol use, particularly for subpopulations of hazardous drinkers with specific treatment goals and high-risk patterns of use.

## Abbreviations

aOR: adjusted odds ratio
AUDIT: Alcohol Use Disorders Identification Test
MSM: men who have sex with men
RDS: respondent-driven sampling

## Data Availability

The datasets generated during and/or analyzed during this study are available from the corresponding author upon request and with applicable institutional review board approval.
